# Race-specific spirometry equations may overestimate asthma control in Black children and adolescents

**DOI:** 10.1186/s12931-023-02505-3

**Published:** 2023-08-17

**Authors:** Allison J. Burbank, Claire E. Atkinson, Andre E. Espaillat, Stephen A. Schworer, Katherine Mills, Jennifer Rooney, Ceila E. Loughlin, Wanda Phipatanakul, Michelle L. Hernandez

**Affiliations:** 1 Division of Allergy & Immunology, Department of Pediatrics, University of North Carolina School of Medicine, Chapel Hill, NC USA; 2 Children’s Research Institute, University of North Carolina, Chapel Hill, NC USA; 3 Division of Pediatric Pulmonology, Department of Pediatrics, University of North Carolina School of Medicine, Chapel Hill, NC USA; 4 Boston Children’s Hospital and Massachusetts General Hospital, Boston, MA USA; 5 Division of Asthma, Allergy and Immunology, Boston Children’s Hospital, Harvard Medical School, Boston, MA USA; 6 5008B Mary Ellen Jones Building 116 Manning Drive, CB #7231, Chapel Hill, NC 27599-7231 USA

**Keywords:** Asthma, Race, Asthma Control Test™, Spirometry, Health disparities

## Abstract

**Background:**

A growing body of evidence suggests that use of race terms in spirometry reference equations underestimates disease burden in Black populations, which may lead to disparities in pulmonary disease outcomes. Data on asthma-specific health consequences of using race-adjusted spirometry are lacking.

**Methods:**

We performed a secondary analysis of 163 children from two observational asthma studies to determine the frequencies of participants with ppFEV1 < 80% (consistent with uncontrolled asthma) or ppFEV1 ≥ 80% using race-specific (GLI-African American or Caucasian) vs. race-neutral (GLI-Global) spirometry and their alignment with indicators of asthma control (Asthma Control Test™, ACT). Comparisons of mean ppFEV1 values were conducted using Wilcoxon matched-pairs signed-rank tests. Two group comparisons were conducted using Wilcoxon rank-sum tests.

**Results:**

Data from 163 children (100 Black, 63 White) were analyzed. Mean ppFEV_1_ was 95.4% (SD 15.8) using race-specific spirometry and 90.4% (16.3) using race-neutral spirometry (p < 0.0001). Among 54 Black children with uncontrolled asthma (ACT ≤ 19), 20% had ppFEV1 < 80% using race-specific spirometry compared to 40% using race-neutral spirometry. In Black children with controlled asthma (ACT > 19), 87% had ppFEV1 ≥ 80% using race-specific compared to 67% using race-neutral spirometry. Children whose ppFEV1 changed to ≤ 80% with race-neutral spirometry had lower FEV1/FVC compared to those whose ppFEV1 remained ≥ 80% [0.83 (0.07) vs. 0.77 (0.05), respectively; p = 0.04], suggesting greater airway obstruction. Minimal changes in alignment of ppFEV1 with ACT score were observed for White children.

**Conclusions:**

Use of race-specific reference equations in Black children may increase the risk of inappropriately labeling asthma as controlled.

## Background

Although race is a social construct with no biological basis, racial adjustment of pulmonary function measurement remains standard practice. Race-specific spirometry reference equations lower the limits of normal lung function in people who identify as Black [1, 2]. Lower lung function in African Americans has long been attributed to genetic differences, including differences in body proportions, without adequately accounting for differences in environmental exposures and social determinants of health. There is growing concern that race-specific spirometry equations mask significant pulmonary disease in Black populations, leading to undertreatment of disease that magnifies racial disparities in health outcomes [3, 4]. For example, use of Global Lung Function Initiative (GLI) African American reference equations significantly underestimated COPD severity in adults [5]. Using linked data from the National Health and Nutrition Examination Survey III (NHANES III) and mortality data, race-specific reference equations predicted lower survival in Black individuals compared to White individuals at a given forced expiratory volume in 1 s (FEV1), suggesting that reduced lung function in Black populations represents clinically significant disease [6]. Similar observations were made with respect to forced vital capacity (FVC) [7]. Additionally, differences in body proportions failed to explain race-based lung function differences [8–11]. These findings provide supporting evidence that lower lung function in Black populations is not normal but instead indicative of higher pulmonary disease burden. These concerns spurred the development of race-neutral approaches to spirometry interpretation, including use of GLI-Other, a composite equation that averages the race-specific equations but is heavily weighted towards Caucasians [1], and GLI-Global, which uses weighting to ensure each racial group contributes equally to the equations, resulting in a wider range of normal lung function [11, 12]. Data on asthma-specific health consequences of using race-adjusted spirometry are lacking.

## Methods

We performed a secondary analysis of data from 163 children (8–18 years) who participated in two observational asthma studies [13, 14] and self-identified as either Black or White to examine the alignment of race-specific and race-neutral spirometry with other clinical indicators of asthma control. Both studies were approved by University of North Carolina Institutional Review Board, and participants provided informed consent/assent. Asthma Control Test™ (ACT) scores were used to categorize asthma as controlled (score ≥ 20) or uncontrolled (≤ 19)[15]. Percent predicted FEV1 (ppFEV1) of ≥ 80% was considered consistent with controlled asthma [16]. ppFEV_1_ was calculated using race-specific (GLI-African American or Caucasian) and race-neutral (GLI-Global) equations (12). Comparisons of mean ppFEV1 values were conducted using Wilcoxon matched-pairs signed-rank test. We determined the frequencies of participants with ppFEV1 < 80% or ppFEV1 ≥ 80% using race-specific vs. race-neutral spirometry and whether ppFEV1 aligned with asthma control defined by ACT scores. Two group comparisons were conducted using Wilcoxon rank-sum test.

## Results

**Fig. 1:**
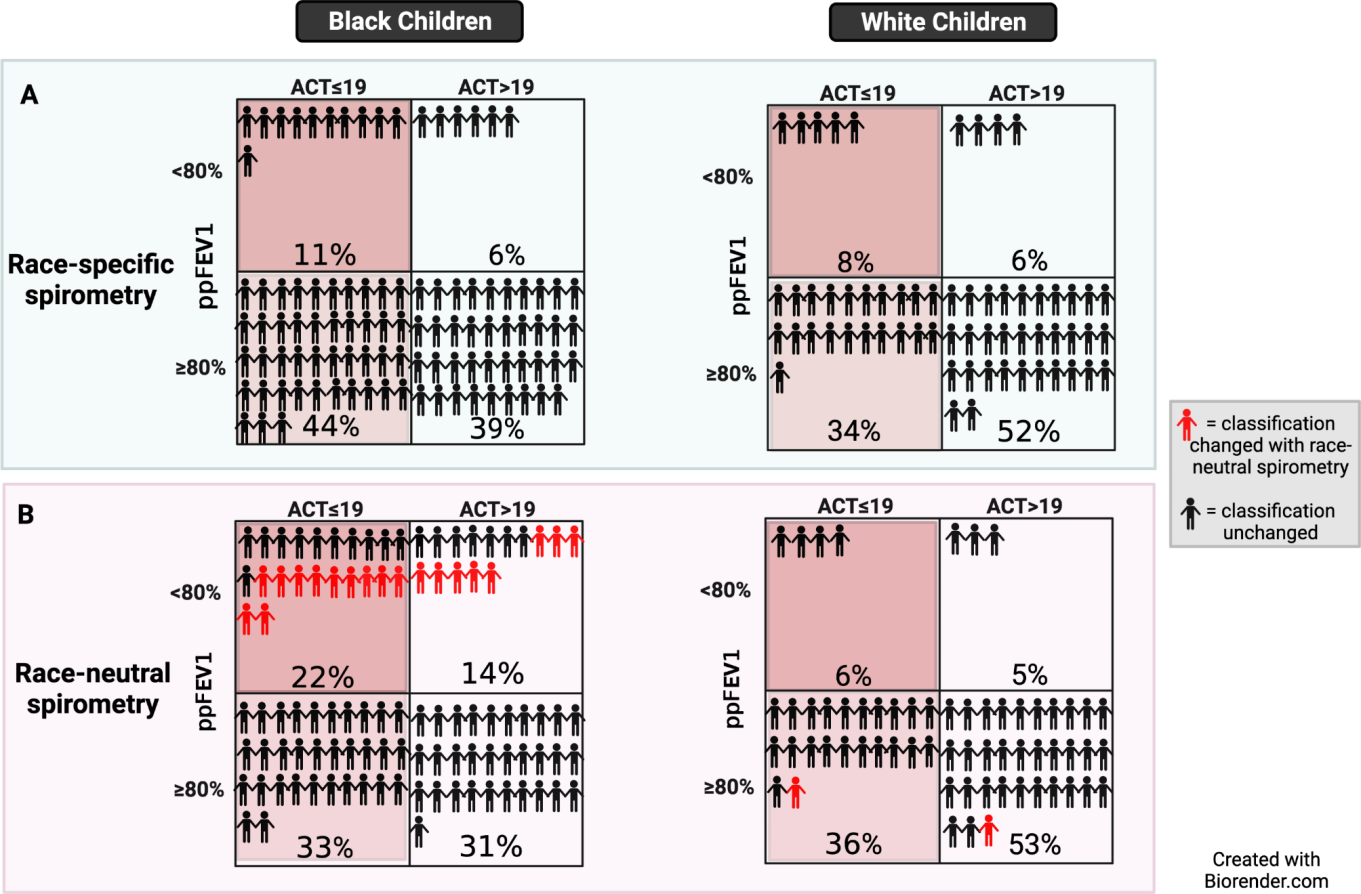
The 2 × 2 table shows the proportions of children with high or low ppFEV1 and high or low ACT score with race-specific and race-neutral spirometry and stratified by race. The figures in red indicate participants whose ppFEV1 categorization changed from controlled to uncontrolled asthma or vice versa

Characteristics of the study population are shown in Table 1. The mean (SD) age of participants was 12.7 (2.5) years, and 61% self-identified as Black. Mean ppFEV_1_ was 95.4% (SD 15.8) using race-specific spirometry and 90.4% (16.3) using race-neutral spirometry (p < 0.0001). ppFEV1 for Black and White children with controlled and uncontrolled asthma are shown in Table 2. Among Black children with uncontrolled asthma (ACT ≤ 19), 11/54 (20%) had ppFEV1 < 80% using race-specific spirometry compared to 22/54 (40%) using race-neutral spirometry (Fig. 1). In Black children with controlled asthma (ACT > 19), 39/45 (87%) had ppFEV1 ≥ 80% using race-specific compared to 31/45 (67%) using race-neutral spirometry. We next examined Black children with ACT > 19 whose ppFEV1 changed from ≥ 80% (reflecting controlled) to < 80% (reflecting uncontrolled) using race-neutral spirometry to assess whether this shift was possibly a reflection of unrecognized airway disease. We compared FEV1/FVC between these children (represented in red in Fig. 1B) and those with ACT > 19 whose ppFEV1 remained ≥ 80% with race-neutral spirometry. Among Black children with high ACT scores, children whose ppFEV1 changed to ≤ 80% (n = 8) with race-neutral spirometry had a significantly lower FEV1/FVC compared to those whose ppFEV1 remained ≥ 80% (n = 31) [0.83 (0.07) vs. 0.77 (0.05), respectively; p = 0.04]. This observation reveals that despite a high ACT score, these children had greater airway obstruction than those whose ppFEV1 remained high, suggesting that the changes in ppFEV1 with race-neutral equations are likely of clinical significance.

**Table 1:** Demographic characteristics of the study population

Characteristic	ACT Study (N = 80)	Active PROMIS study (N = 83)	Total Sample (N = 163)
Age in years, mean (SD)	13.7 (1.8)	11.7 (2.6)	12.7 (2.5)
Sex, % female	49	49	49
Race, N(%) Black White	53 (66) 27 (34)	47 (57) 36 (43)	100 (61) 63 (39)
Ethnicity – Hispanic/Latinx, N(%)	1 (1)	10 (12)	11 (7)
ACT score, mean (SD)	20 (4)	18 (5)	19 (5)
ppFEV1, mean (SD) Race-specific Race-neutral	95.5 (16.6) 90.5 (16.7)	95.3 (15) 90.3 (16.1)	95.4 (15.8) 90.4 (16.3)
FEV1/FVC, mean (SD)	0.80 (0.09)	0.79 (0.08)	0.80 (0.08)

**Table 2:** Percent predicted FEV1 using race-specific and race-neutral spirometry stratified by asthma control status

	Uncontrolled Asthma (ACT ≤ 19) (n = 81)		Controlled Asthma (ACT > 19) (n = 82)	
**ppFEV1**, mean (SD)				
	**Black** **(n = 54)**	**White** (n = 26)*	**Black** (n = 45)*	**White** **(n = 36)**
*Race-specific spirometry*	94.1 (16.7)	96.1 (16.2)	96.7 (17)	95.1 (12.7)
*Race-neutral spirometry*	84.3 (14.8)	100 (16.6)	86 (15.7)	98 (13.1)

*spirometry data were missing from 2 participants (1 White, 1 Black)

## Discussion

We observed that use of race-neutral spirometry resulted in a doubling of the number of Black children with abnormally low lung function compared to race-specific spirometry. Of these children, 58% had an ACT score ≤ 19, consistent with uncontrolled asthma. Those with low ppFEV1 despite high ACT score had significantly greater airway obstruction reflected in FEV1/FVC, a measure that is not reliant on race/ethnicity terms, than Black children with high ppFEV1 and high ACT score. We observed minimal impact on White children’s asthma control classification with use of race-neutral spirometry, with only one participant with low ACT score going from ppFEV1 < 80% to ≥ 80%. We interpret our findings as supportive of the theory that use of race-specific equations may lead to under-recognition of uncontrolled asthma in Black children.

Approaching race and ethnicity in lung function testing is a complex issue. While GLI global equations have certain advantages over GLI-Other and race-specific equations, they are not truly race-neutral, and many global populations are not represented [11]. Broadening the limits of “normal” lung function will mean that some true disease will be mislabeled as normal and in other cases disease may be over-diagnosed [17]. We recognize that the implications of over-diagnosing pulmonary disease are serious and can have a significant impact on insurability and eligibility for medical procedures and employment opportunities. Globally representative studies inclusive of health outcomes are needed to determine the optimal way to define “normal” lung function. In addition to the small size of our sample, we acknowledge a limitation in our study was the use of ACT scores to define asthma control. Though widely accepted in clinical care and research, we previously reported that the ACT may have reduced validity in Black adolescents, and the suggested cut point score of 19 may be too low to adequately capture uncontrolled asthma in this population [13]. Therefore, we may have underestimated the proportion of the study population whose uncontrolled asthma was masked by use of race-specific reference equations. Additionally, ACT measures symptoms over time such that FEV1 often does not correlate well with asthma symptoms, which may have affected our results.

## Conclusions

In conclusion, use of race/ethnicity-specific terms in spirometry reference equations in Black children may increase the risk of inappropriately labeling asthma as “controlled”, which if undertreated could increase the risk of long-term negative effects on lung health. Identification of optimal reference equations, including the performance of multiracial and race-neutral equations, are needed to improve detection of clinically significant disease to avoid perpetuating existing asthma-related health disparities.

## Data Availability

The datasets used and/or analyzed during the current study are available from the corresponding author on reasonable request.

## Abbreviations

(GLI): Global Lung Function Initiative
(NHANES III): National Health and Nutrition Examination Survey III
(FEV1): Forced expiratory volume in 1 s
(FVC): Forced vital capacity
(ACT): Asthma Control Test™
(ppFEV1): Percent predicted FEV1

## References

[1] Quanjer PH, Stanojevic S, Cole TJ, Baur X, Hall GL, Culver BH (2012). Multi-ethnic reference values for spirometry for the 3-95-yr age range: the global lung function 2012 equations. Eur Respir J.

[2] Hankinson JL, Odencrantz JR, Fedan KB (1999). Spirometric reference values from a sample of the general U.S. population. Am J Respir Crit Care Med.

[3] Akinbami LJ, Moorman JE, Bailey C, Zahran HS, King M, Johnson CA et al. Trends in asthma prevalence, health care use, and mortality in the United States, 2001–2010. NCHS Data Brief. 2012(94):1–8. 22617340

[4] Pate CA, Zahran HS, Qin X, Johnson C, Hummelman E, Malilay J (2021). Asthma surveillance - United States, 2006–2018. MMWR Surveill Summ.

[5] Baugh AD, Shiboski S, Hansel NN, Ortega V, Barjaktarevic I, Barr RG (2022). Reconsidering the utility of race-specific lung function prediction equations. Am J Respir Crit Care Med.

[6] McCormack MC, Balasubramanian A, Matsui EC, Peng R, Wise RA, Keet CA, Race. Lung function and long-term mortality in the National Health and Examination Survey III. Am J Respir Crit Care Med. 2021.10.1164/rccm.202104-0822LE34597248

[7] Burney PG, Hooper RL (2012). The use of ethnically specific norms for ventilatory function in african-american and white populations. Int J Epidemiol.

[8] Lum S, Bountziouka V, Sonnappa S, Wade A, Cole TJ, Harding S (2015). Lung function in children in relation to ethnicity, physique and socioeconomic factors. Eur Respir J.

[9] Harik-Khan RI, Fleg JL, Muller DC, Wise RA (2001). The effect of anthropometric and socioeconomic factors on the racial difference in lung function. Am J Respir Crit Care Med.

[10] Harik-Khan RI, Muller DC, Wise RA (2004). Racial difference in lung function in african-american and white children: effect of anthropometric, socioeconomic, nutritional, and environmental factors. Am J Epidemiol.

[11] Bhakta NR, Bime C, Kaminsky DA, McCormack MC, Thakur N, Stanojevic S (2023). Race and ethnicity in pulmonary function test interpretation: an official american thoracic Society Statement. Am J Respir Crit Care Med.

[12] Bowerman C, Bhatka NR, Brazzale D, Cooper BR, Cooper J, Gochicoa-Rangel L et al. A race-neutral Approach to the interpretation of lung function measurements. Am J Respir Crit Care Med. 2022. 10.1164/rccm.202205-0963OC 36383197

[13] Burbank AJ, Todoric K, Steele P, Rosen J, Zhou H, Frye M (2018). Age and african-american race impact the validity and reliability of the asthma control test in persistent asthmatics. Respir Res.

[14] Hernandez ML, Lucas N, Mann C, Lin L, Burbank AJ, Brown J (2021). Association of step count with PROMIS pediatric health-related quality of life measures in children and adolescents with persistent asthma. J Allergy Clin Immunol Pract.

[15] Schatz M, Sorkness CA, Li JT, Marcus P, Murray JJ, Nathan RA (2006). Asthma Control Test: reliability, validity, and responsiveness in patients not previously followed by asthma specialists. J Allergy Clin Immunol.

[16] 2020 Focused Updates to the Asthma Management Guidelines. A report from the National Asthma Education and Prevention Program Coordinating Committee Expert Panel Working Group. National Heart Lung and Blood Institute; 2020. 10.1016/j.jaci.2020.10.003 PMC7924476 33280709

[17] Bhakta NR, Kaminsky DA, Bime C, Thakur N, Hall GL, McCormack MC (2022). Addressing race in pulmonary function testing by aligning intent and evidence with practice and perception. Chest.

